# Construction of a Novel Liver-Targeting Fusion Interferon by Incorporation of a *Plasmodium* Region I-Plus Peptide

**DOI:** 10.1155/2014/261631

**Published:** 2014-01-19

**Authors:** Xuemei Lu, Xiaobao Jin, Yanting Huang, Jie Wang, Juan Shen, Fujiang Chu, Hanfang Mei, Yan Ma, Jiayong Zhu

**Affiliations:** ^1^Guangdong Provincial Key Laboratory of Pharmaceutical Bioactive Substances, Guangdong Pharmaceutical University, 280 Wai Huan Dong Road, Guangzhou Higher Education Mega Center, Guangzhou 510006, China; ^2^School of Basic Courses, Guangdong Pharmaceutical University, 280 Wai Huan Dong Road, Guangzhou Higher Education Mega Center, Guangzhou 510006, China

## Abstract

Interferon alpha (IFN **α**) exerts a multiplicity of biological actions including antiviral, immunomodulatory, and antiproliferative effects. Administration of IFN **α** is the current treatment for chronic hepatitis B; however, therapy outcome has not been completely satisfactory. The systemic effects of IFN **α** may account for its low *in vivo* biological activity and multiple adverse events. The purpose of this study was to design a novel liver-targeting fusion interferon (IFN-CSP) by fusing IFN **α**2b with a *Plasmodium* region I-plus peptide, thus targeting the drug specifically to the liver. The DNA sequence encoding IFN-CSP was constructed using improved splicing by overlapping extension-PCR method, and then cloned into the pET-21b vector for protein expression in *E. coli* BL21 (DE3). The recombinant protein was expressed as a His-tagged protein and purified using a combination of Ni affinity and HiTrap affinity chromatography at a purity of over 95%. The final yield of biologically active IFN-CSP was up to 270 mg/L culture. The purified recombinant protein showed anti-HBV activity and liver-targeting potentiality *in vitro*. These data suggests that the novel fusion interferon IFN-CSP may be an excellent candidate as a liver-targeting anti-HBV agent.

## 1. Introduction

Interferons alpha (IFN *α*) are a multigene family of inducible cytokines whose major therapeutic applications are based on their antiviral, immunomodulatory, and antiproliferative functions [1, 2]. IFN *α*2b is the first one used in the clinic for the treatment of chronic hepatitis B and C and several cancers such as melanoma, AIDS-related Kaposi's sarcoma, chronic myeloid lymphoma, and angioblastoma [3]. However, because interferon does not have organ-specific affinity, patients may not always achieve a therapeutic effect [4]. Additionally, the drug has significant side effects, such as serious depression, fatigue, influenza-like syndrome, myalgia, bone marrow suppression, alopecia, and autoimmune disorders. These side effects can be severe which leads to a significant proportion of patients discontinuing treatment [5].

Targeting of drugs to specific cells or tissues is an attractive strategy in terms of improving the safety and efficacy of the therapeutic [6]. In particular, because of the localization and viral replication in HBV infection mainly occur in the liver, there have been numerous efforts by various scientists to selectively deliver therapeutic drugs to the liver using different approaches. For example, it has been shown that conjugates of antiviral drugs, with lactosaminated human serum albumin, resulted in accumulation of these antiviral drugs in the liver cells, increasing the effectiveness of the drugs in comparison with the free drugs [7]. Others have used glycosylated lipoproteins or arabinogalactan as liver-accumulating carriers for targeted delivery of antiviral drugs to the liver, potentially retaining efficacy with a reduced dose [8]. These studies support the general concept that targeted delivery of antiviral drugs to the liver increases the efficacy of these drugs in the treatment of viral liver infections, while at the same time it decreases potential side effects in other nontarget tissues. However, the choice of targeting moieties and carrier needs optimization to improve drawbacks associated with variability in affinity and density seen with the asialoglycoprotein receptors [8] or to reduce carrier-related side effects, such as increased alkaline phosphatase levels seen with lactosaminated albumin [7].

Numerous microorganisms have developed highly effective organ specific targeting strategies for host invasion [9]. In nature, the protozoans *Plasmodium falciparum* exhibit a remarkably effective liver-targeting strategy [10]. When introduced into the circulation, the sporozoite stage of the organism is cleared by the liver within minutes. The rapid and specific targeting of *Plasmodium* sporozoite to liver is attributed to the circumsporozoite protein (CSP), which is present on the surface of *Plasmodium* sporozoite [11]. CSP is approximately 400 amino acids long organized into three domains: the N-terminal domain containing a conserved KLKQP motif named “region I”, a highly repetitive central domain, and a C-terminal domain containing another conserved sequence named “region II” [12]. In addition to the conserved region I KLKQP sequence, the N-terminal region also contains upstream from region I, two consensus heparin sulfate binding sequences. Peptides containing both the conserved region I amino acids and two consensus heparin binding sequences upstream from region I have been named “region I-plus” [13]. Ancsin and Kisilevsky have demonstrated [14] that only the region I-plus sequence binds strongly to affinity columns of heparin and heparan sulfate. In addition, only the region I-plus peptide is able to inhibit binding of recombinant CSP to heparin-Sepharose. It is proposed that the region I-plus sequence is primarily responsible for the liver specificity of CSP [14]. Thus, incorporation of the *Plasmodium* region I-plus peptide may be a promising strategy for the development of liver-targeting drug.

To our knowledge, production of a liver-targeting IFN-CSP by genetic engineering has not been reported. In the present study, a novel hybrid gene combining IFN *α*2b with *Plasmodium* region I-plus peptide was designed and expressed in *E. coli*. We further investigated whether the fusion protein IFN-CSP reserves anti-HBV activity and liver-targeting function as its parental peptides.

## 2. Materials and Methods

### 2.1. Bacterial Strains, Plasmids, and Culture Media

*E. coli* strain DH5*α* (Novagen, USA) and BL21 (DE3; Novagen, USA) were used as the host for gene manipulation and expression of fusion protein, respectively. pMD20-T (Takara, Japan) and pET-21b (Novagen, USA) were applied for gene cloning and expression, respectively. Luria-Bertani (LB) medium was employed for bacterial growth and protein production.

### 2.2. Construction of Liver-Targeting Fusion Interferon Gene by SOE-PCR

**Figure 1:**
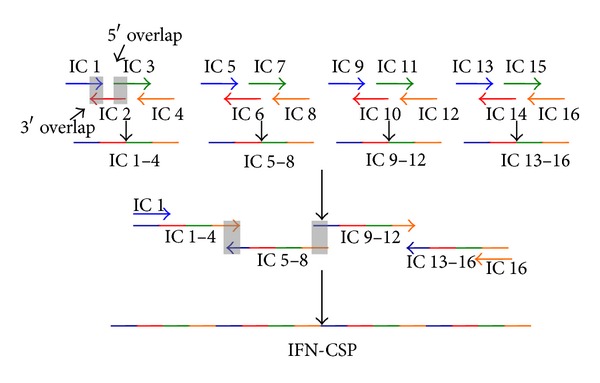
Schematic diagram of the gene synthesis method. The target DNA is dissected into oligos of between 29 and 58 nt long. Each four adjacent primers were mixed in a separate tube; after the first PCR, fragments overlap adjacent ones by up to 175 bp. In the overlap extension PCR step, the terminal fragments can be easily extended to full length.

The full-length liver-targeting fusion interferon gene was constructed using improved splicing by overlapping extension-PCR (SOE-PCR) method [15, 16]. The nucleotide sequences of IFN *α*2b (GenBank accession no. NP_000596) and CSP (GenBank accession no. X15363) were obtained from NCBI. Oligonucleotides for gene assembly were designed using DNA Star (DNA Star, Inc.). The sequences of the oligonucleotides with 19 bp complementary overlapping sequence each other are presented in Table 1. The process of SOE-PCR is depicted in Figure 1. In step 1, every four consecutive oligonucleotides were mixed together in a separate tube, with the outer two oligonucleotides at five times molar excess to the inner ones. PCR was performed as follows: thirty cycles of 10 s at 98°C (denaturation) and 68°C for 30 s (annealing and extension). In step 2, the resulting fragments were mixed together, and PCR was performed as follows: ten cycles of 10 s at 98°C and 68°C for 40 s. The third step was “PCR amplification”, using IC-1 and IC-16 (Table 1) as out-primers, and the full-length gene was generated by PCR. The PCR conditions were as follows: thirty cycles of 10 s at 98°C and 68°C for 40 s.

### 2.3. Construction of Expression Plasmids IFN-CSP/pET-21b

**Figure 2:**
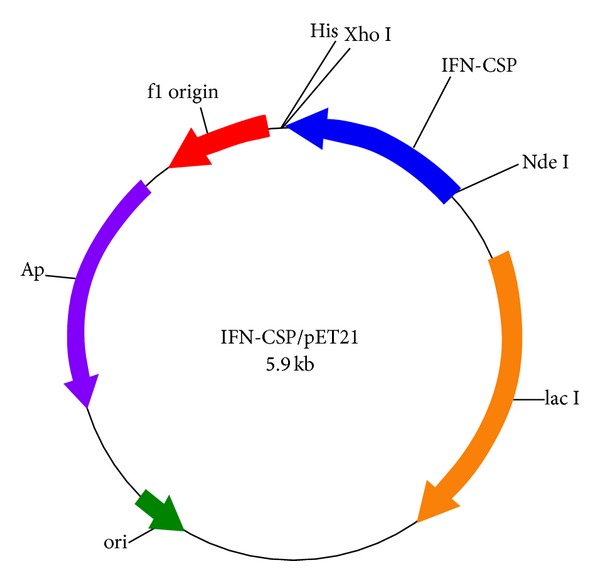
Schematic representation of expression vector IFN-CSP/pET-21b.

The full length IFN-CSP gene was recovered using the Gel Extraction Kit (Tiangen, China) and cloned into the pMD20-T vector in *E. coli* DH5a according to the protocol described by the manufacturer (Takara). The generated recombinant plasmids IFN-CSP/pMD20-T was digested by Nde I and Xho I restriction enzymes and the restriction fragment was cloned into expression vector pET-21b digested with same restriction enzymes (Figure 2). The resulting expression plasmid IFN-CSP/pET-21b was finally transformed into *E. coli* BL21 (DE3) for protein expression.

### 2.4. Protein Expression and Purification

*E. coli* BL21 (DE3) harboring the IFN-CSP/pET-21b plasmids were grown in Luria-Bertani (LB) medium containing 100 **μ**g/mL ampicillin, incubated at 37°C to reach the midlog phase. Then, the recombinant IFN-CSP was induced by adding isopropylthio-D-galactoside (IPTG) to a final concentration of 0.8 mM and incubating for an additional 6 h. Cells were harvested by centrifugation and lysed by ultrasonication in an ice bath for 10 min. The precipitate was recovered by centrifugation (12,000 g, 8 min, 4°C). The inclusion bodies were dissolved in 6 M guanidine hydrochloride (GuHCl) (containing 50 mM Tris-HCL buffer, 2.5 mM DTT, pH 8.0), and the soluble IFN-CSP was purified by Ni affinity chromatography according to the protocol of HisTrap kit (GE healthcare, USA). The purified proteins were collected and refolded by dialysis with the buffer systems (50 mM Tris-HCl buffer, 2 mM glutathione reduced, 0.2 mM glutathione oxidized, pH 8.0), containing decreasing concentrations of the denaturing agent (4, 2, 1, and 0.5 M GuHCl) and finally with Tris-HCl buffer (pH 7.4). The sample was applied to HiTrap affinity column (GE healthcare, USA) equilibrated with above Tris-HCl buffer. Bound proteins were eluted by a linear gradient of 0.1–2 M sodium chloride. The last step of purification was dialysis to remove sodium chloride. Protein concentration was determined by the Bradford method. The purified IFN-CSP was lyophilized and stored at −80°C.

Total proteins and purified IFN-CSP were analyzed by 15% sodium dodecyl sulfate polyacrylamide gel electrophoresis (SDS-PAGE). The percentage fraction of proteins was assessed by densitometric using Gel-Pro analyzer Version 4.5 software. The antigenicity of fusion protein was characterized by Western blot analysis. Protein bands were transferred from the gel to polyvinylidene difluoride (PVDF) membranes using semidry electrophoretic transfer (Bio-Rad, USA). Recombinant proteins were detected by using goat polyclonal anti-human IFN *α* antibody (1 : 500; incubated for 2 h; Santa Cruz Biotechnology, USA). The final purity of recombinant IFN-CSP was assessed by reverse phase high-performance liquid chromatography (RP-HPLC) on a C18 column (250 × 4.6 mm, 5 *μ*m, and 300 Å, Agilent, USA) in an analytical Alliance HPLC System (Waters, USA). The gradient was prepared using solvent A (0.1%, v/v, trifluoroacetic acid in water) and solvent B (0.1%, v/v, trifluoroacetic acid in acetonitrile). Elution was performed with a linear gradient of 20–80% solvent B in 60 min at a flow rate of 1 mL/min. The protein was detected by its absorption at 220 nm. Peak areas were integrated using Empower Chromatography Data software (Waters).

### 2.5. LPS Content

Recombinant IFN-CSP was further purified by using a polymyxin B column (Bio-Rad, USA) to remove endotoxin. The LPS content of the sample was detected by the chromogenic limulus amoebocyte lysate assay (Associates of Cape Cod, USA).

### 2.6. *In Vitro* Specific Biological Activity

The antiviral activity of IFN-CSP was determined by its ability to inhibit the cytopathic effect caused by vesicular stomatitis virus (VSV) on human amniotic cells (WISH) according to China Biologicals Requirements. The effect was determined by measuring the cellular uptake of crystal violet dyes at 570 nm. Each sample was run in five times. All titres were reported in IU/mL.

The anti-HBV activity of IFN-CSP was measured as the ability to inhibit the secretion of HBV DNA from HepG 2.2.15 cells. The HBV-DNA integrated hepatoma cell line HepG 2.2.15 which produces infectious viral particles, was grown in complete DMEM (Gibco-BRL, CA) containing 380 **μ**g/mL G418 antibiotic (Sigma, MO), 100 mg/mL streptomycin, 100 units/mL penicillin, and 10% FBS (Hyclone, Thermo Fisher, PA). Cells were cultured at 37°C in a humidified 5% CO_2_ atmosphere. The HepG 2.2.15 cells (2 × 10^4^/well) were seeded in 24-well plates. After 24 h of incubation, the culture medium was replaced by fresh medium (2% FBS) with IFN *α*2b (at final a concentration of 10^3^ U/l, *n* = 5) or recombinant IFN-CSP (at final a concentration of 10^3^ U/l, *n* = 5) for 3, 6, and 9 days. The same volume of culture medium without drugs was added to the control wells (*n* = 5). The culture medium was replaced every 3 days. The supernatants were collected on the 3rd, 6th, and 9th days. HBV DNA was extracted from culture supernatants and quantified using a commercially available real-time fluorescence quantitative PCR kit (FQ-PCR, Da-An Gene Co., Guangzhou, China) based on the TaqMan technology. FQ-PCR was run on a LightCycler instrument (Bio-Rad, Mannheim, Germany). Data shown represent the mean values (±S.D.) based on three independent experiments. One-Way ANOVA was used for statistical analyses.

### 2.7. *In Vitro* Application of IFN-CSP to Fixed Slices from Normal Animal

Balb/c mice, approximately 20–25 g, were purchased from the Center for Experimental Animals, Guangdong Province (Guangzhou, China). All research procedures involving mice were approved by the Guangdong Pharmaceutical University Animal Care and Use Committee. Mice were deeply anesthetized with sodium pentobarbital (100 mg/kg; intraperitoneal) and euthanized by perfusion through the heart with saline followed by 50 mL of 4% paraformaldehyde fixative at a flow rate of 5 mL/min, and tissues were collected and cut into 2-3 mm thick blocks and postfixed in 4% paraformaldehyde for 1 h and then in 30% sucrose solution overnight for cryoprotection. Tissue blocks were cut into 10–12 **μ**m thick sections using a Leica cryostat. Sections were preincubated for 1 h with 3% normal goat serum. Sections were then incubated overnight at 4°C with IFN-CSP solution. The following day, sections were washed three times with Tris buffer and incubated for 2 h with primary antibody (1 : 200; goat polyclonal anti-human IFN *α* antibody, from Santa Cruz Biotechnology, USA) at room temperature. After the incubation, the sections were washed three times with Tris buffer and incubated for 30 min in darkness with Alexa Fluor 488-conjugated donkey anti-goat IgG (1 : 1000; Invitrogen-life Technologies, Carlsbad, CA). Once secondary antibody was removed, the sections were rinsed in Tris buffer and stained with 4′,6-diamidino-2-phenylindole (DAPI), a fluorescent nuclear stain useful for studying nuclear morphology and tissue architecture. The slices were washed three times and coverslipped with Vectashield (Vector Labs, Burlingame, CA). Immunofluorescence analyses were performed with a Leica fluorescence microscope. Immunocytochemical controls included tissue processed without IFN-CSP solution or primary antibodies or secondary antibodies; no staining was obtained in these cases.

## 3. Results

### 3.1. Construction of Expression Plasmids

**Figure 3:**
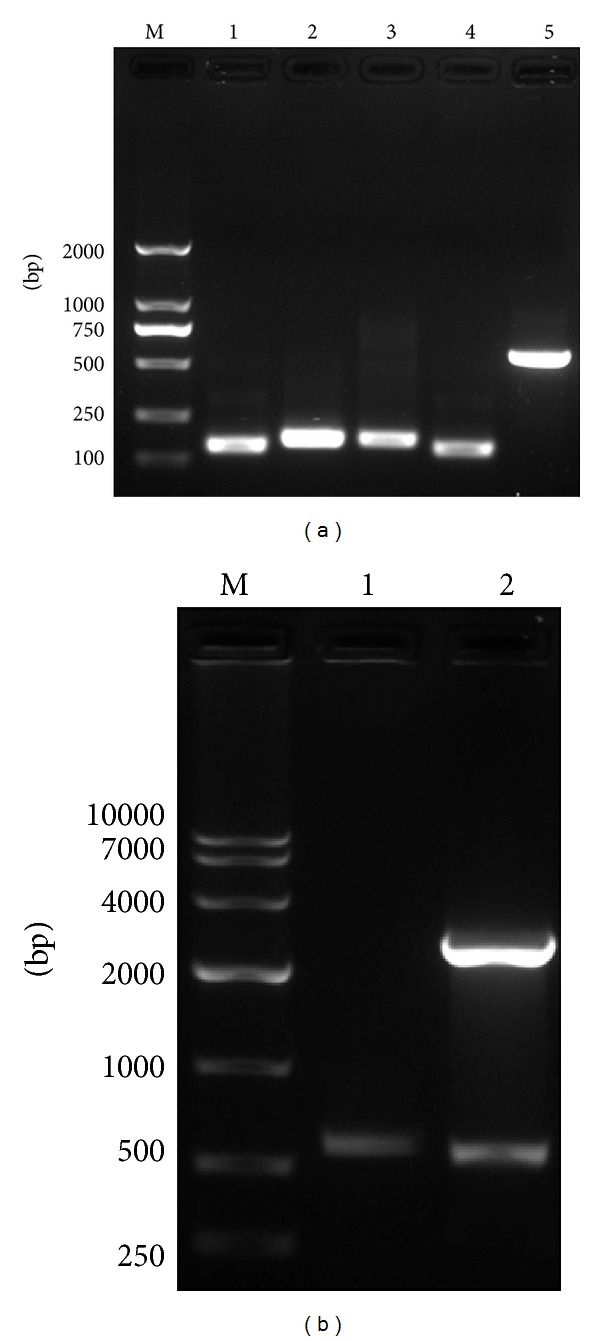
Construction of fusion gene IFN-CSP and screening of recombinant plasmids containing fusion gene. (a) Fusion gene IFN-CSP was constructed by SOE-PCR. Lane M: DNA molecular weight marker. Lane 1–4: first PCR was performed for fusion gene assembly using eight pairs of oligonucleotides with different concentrations as templates to give PCR products ranging 132–175 bp in size. Lane 5: target amplification fragment of fusion gene IFN-CSP. (b) Screening of recombinant plasmids containing IFN-CSP gene by colony PCR and restriction endonuclease analysis. Lane M: DNA molecular weight marker, Lane 1: PCR products of recombinant plasmids, and Lane 2: Recombinant plasmids digested with Nde I/Xho I.

The full-length IFN-CSP gene was obtained using SOE-PCR method, and in Figure 3(a), lane 5 shows a single distinct DNA band of about 550 bp in size which was close to the theoretical size of IFN-CSP, 552 bp. The resulting IFN-CSP gene fragment was cloned in-frame with the 6His tag sequence in the expression vector pET-21b to construct recombinant expression plasmids IFN-CSP/pET-21b. After PCR screening and restriction endonuclease analysis, the recombinant plasmid gave the predicted size (Figure 3(b)). The sequencing data verified that the recombinant plasmid carried IFN-CSP.

### 3.2. Recombinant Expression and Purification of IFN-CSP

**Figure 4:**
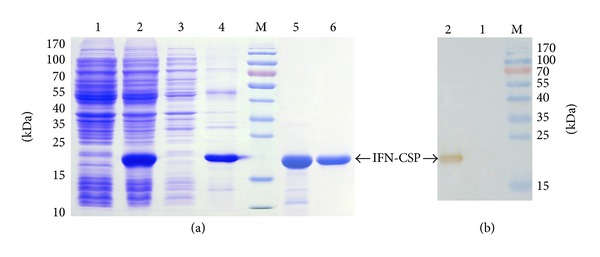
Analysis of fusion protein by SDS-PAGE and Western blot. (a) Expression and purification of IFN-CSP. Lane M: protein molecular weight marker. Lanes 1 and 2: total proteins of *E. coli* BL21/pET-21b-IFN-CSP before and after induction. Lanes 3 and 4: supernatant and precipitation after ultrasonication and centrifugation. Lane 5: purified IFN-CSP using Ni affinity chromatography. Lane 6: Purified IFN-CSP using HiTrap affinity chromatography. (b) IFN-CSP was analyzed by SDS-PAGE, transferred to PVDF membrane, and detected by goat polyclonal anti-human IFN *α* antibody. Lane M: protein molecular weight marker. Lanes 1 and 2: total proteins of *E. coli* BL21/pET-21b-IFN-CSP before and after induction.

**Figure 5:**
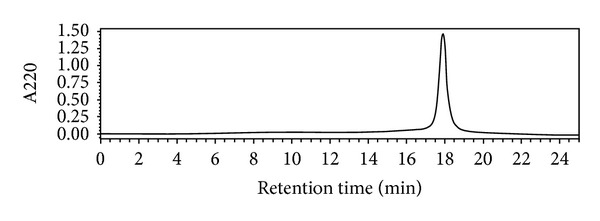
RP-HPLC analysis of purified fusion protein IFN-CSP with a C18 column.

For protein expression, the recombinant plasmids IFN-CSP/pET-21b were transformed into *E. coli* BL21 (DE3). After IPTG induction, the recombinant IFN-CSP was insoluble and formed inclusion bodies (Figure 4(a), lane 2), accounting for 39.2% of the total insoluble protein as assessed by densitometric scanning. The molecular weight (MW) of IFN-CSP was shown to be approximately 21.5 kDa, as expected. Western blot analysis resulted in a specific signal at 21.5 kDa with goat polyclonal anti-human IFN *α* antibody, whereas no cross-reaction occurred in the total protein from *E. coli* BL21/pET-21b-IFN-CSP cells before induction, confirming that the IFN-CSP protein specifically reacted with the IFN *α* antibody (Figure 4(b)). Using a combination of Ni affinity and HiTrap affinity chromatography, IFN-CSP was purified to over 95% homogeneity with no degradation as demonstrated by SDS-PAGE (Figure 4(a), lane 6) and RP-HPLC analysis (Figure 5). These procedures were highly efficient in producing pure IFN-CSP; approximately 270 mg of the pure recombinant IFN-CSP was obtained from 1 L of *E. coli* culture.

### 3.3. *In Vitro* Specific Biological Activity

The LPS content in the recombinant protein was less than 0.5 Eu per mg proteins. Antiviral analysis in the cytopathic effect inhibition assay using the WISH/VSV system showed that IFN-CSP had ability to inhibit the cytopathic effect caused by VSV on WISH cells (1.2 × 10^8^ U/mg).

**Figure 6:**
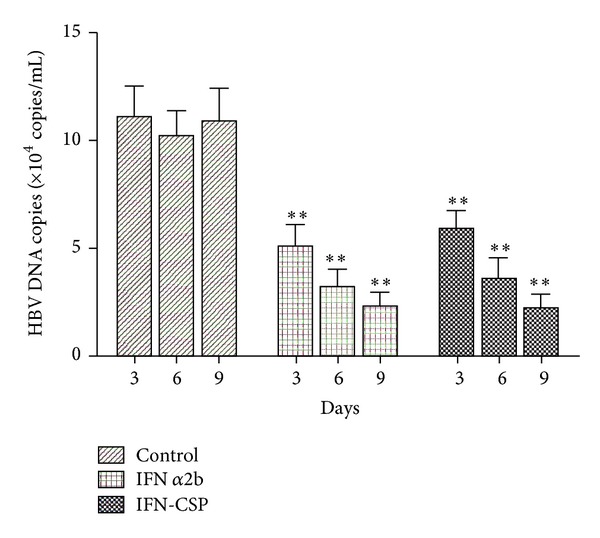
Inhibition of HBV DNA replication by IFN *α*2b and IFN-CSP in HepG 2.2.15 cells. The HBV DNA in supernatants of the HepG 2.2.15 cells was harvested and analyzed by real-time PCR. The copies of the HBV DNA from the HepG 2.2.15 cells were calculated based on their Ct value and the standard curves. Data shown represent the mean values (±S.D.) based on three independent experiments. **P < 0.01 as compared with the control group.

To confirm the anti-HBV activity of IFN-CSP in HepG 2.2.15 cells, the HBV DNA levels were evaluated after treatment. The abundance of HBV-DNA released into the culture medium of IFN-CSP group and native IFN *α*2b group was significantly decreased compared with that of the control group; Figure 6 indicated that they have similar inhibition effects on HBV DNA replication in HepG 2.2.15 cells.

### 3.4. IFN-CSP Specific Targeting to Liver Tissue

**Figure 7:**
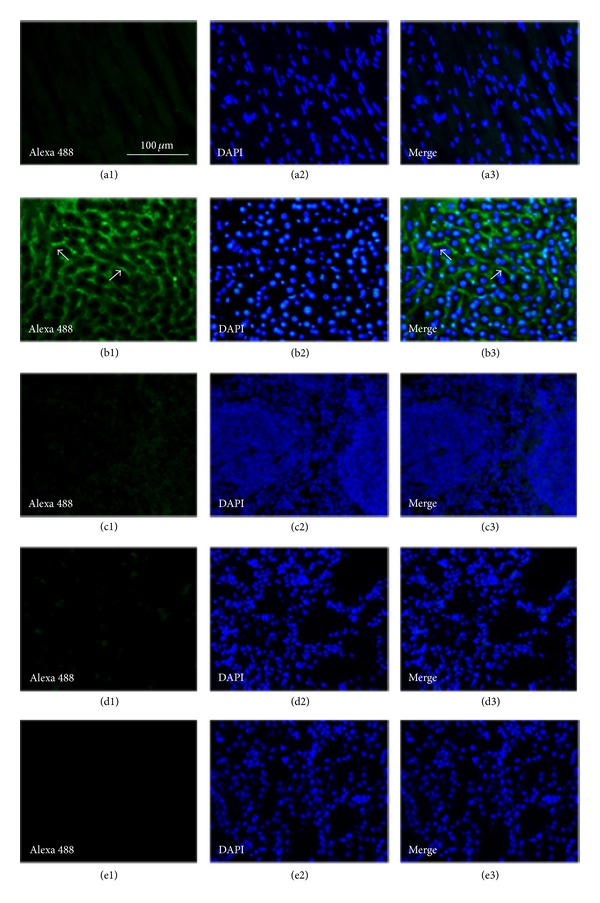
Fluorescence photomicrographs of tissue from an adult BALB/c mouse after being incubated with IFN-CSP solution. (a) Heart. (b) Liver. (c) Spleen. (d) Lung. (e) Kidney. 1: green fluorescent labeling of IFN-CSP stained by anti-IFN antibody. 2: blue nuclear stained with DAPI. 3: merged images of 1 and 2. Note the distinct green fluorescent labeling of IFN-CSP in liver, but not in heart, spleen, lung, or kidney. Bar, 100 **μ**m, is the same for all photomicrographs. Arrows indicate two examples of sinusoidal capillary profiles.

To determine whether IFN-CSP was able to specificly target liver, IFN-CSP solution was administered by direct application to tissue slices.  Figure 7 presents examples of fluorescent IFN-CSP labeling of tissues after incubation. The photomicrograph in Figure 7(b) shows that fluorescence appears along the sinusoidal borders and along the basolateral region of the hepatocytes adjacent to the sinusoids. In contrast, neither the heart nor the kidney and lung (Figures 7(a), 7(c), 7(d), and 7(e)) displayed any IFN-CSP labeling of cell.

## 4. Discussion

As the first one used in the clinic for the treatment of hepatitis B, therapy outcome of interferon *α*2b has not been completely satisfactory. In the present study, we report the first successful attempt to design and construct a novel liver-targeting fusion interferon IFN-CSP by fusing human IFN *α*2b with a CSP region I-plus peptide derived from* Plasmodium*. The purified recombinant protein showed anti-HBV activity and liver-targeting potentiality, providing a promising therapeutic agent for hepatitis B therapy.

To explore a pharmaceutical potential of liver-targeting interferon, a scalable and cost-effective method to produce a large quantity of active proteins is required. Two common strategies have been employed to construct liver-targeting interferon: physical/chemical modification (e.g., macromolecular carriers bearing) and gene fusion (e.g., antibody fusion). Compared with physical/chemical modification, gene fusion technology has the advantages of more homogeneous product and lower cost, and thus this is promising for the development of liver-targeting protein drugs. In the present study, we report the first successful attempt to fuse a *Plasmodium* region I-plus peptide with IFN *α*2b using gene fusion technology. Gene cloning has long been the basis for recombinant gene projects. However, the gene cloning step normally requires the presence of template DNA, which is not always readily available [17]. Compared to cloning the natural cDNA sequence by reverse transcriptase PCR (RT-PCR), gene synthesis does not require a template and does not suffer from limitations of resources, which is particularly relevant for rare and endangered species or if the gene is expressed at an extremely low level. However, full length gene synthesis is highly expensive [15, 16]. In addition, long length hinders successful gene synthesis. In this study, we describe an improved SOE-PCR method for low-cost gene synthesis to effectively reduce the length of oligonucleotides used for the gene assembly process to under 58 nt. The final full-length DNA product without any DNA sequence errors demonstrated that the improved SOE-PCR method is reliable.

The final full-length gene was transformed into a prokaryotic expression system to express a large quantity of biologically active proteins. There are four reasons for using *E. coli* as an expression system: (1) *E. coli* expression system is low cost and high yield, (2) genetically modified *E. coli* is safe for large-scale production, (3) IFN-CSP is a nonglycosylated protein, and (4) the gene encoding for IFN-CSP is intronless. Several fusion proteins such as novel dual-functional protein LL-37-haFGF [18], fusion protein Mdc-hly [19], and fusion interferon SAK-IFN [18, 20] have been successfully expressed in *E. coli*. There are also several reports attempting over expressed IFN *α*2b gene in *E. coli* in recent years. Retnoningrum et al. constructed a synthetic open reading frame encoding human IFN *α*2b for over expression as a thioredoxin-his-tag fusion protein in *E. coli* BL21. The rhIFN*α*2b fusion protein was isolated from inclusion bodies (IB), renatured, and purified with a yield of 3.46 mg/L [21]. Valente et al. used *E. coli* codon optimized artificial gene that produced 16.6 mg/L of IFN *α*2b [22]. Rabhi-Essafi et al. merged the IFN *α*2b cDNA with the glutathione S-transferase (GST) coding sequence. The results show the production of soluble and functional IFN *α*2b at a yield of 100 mg/L [23]. Genetically modified by single-step assembly method for *E. coli* codon usage, Neves et al. reported that they obtained −210 mg/L for His-IFN *α*2b and −75 mg/L for without histidine tag [24]. In the present study, the fusion protein was successfully expressed and the final yield of IFN-CSP was up to 270 mg per L of *E. coli* culture. IFN-CSP was easily purified using two affinity chromatographic steps. Ni^2+^-based immobilized metal ion affinity chromatography was used for the capture of the fusion protein under denaturing conditions, whereas HiTrap affinity chromatography was applied to polish the IFN-CSP after the recovering of the recombinant protein. The purity of recombinant IFN-CSP was higher than 95%, as determined by SDS-PAGE and RP-HPLC.

The inhibitory effects of IFN-CSP on HBV replication and cytopathic effect caused by VSV were investigated. Results showed that IFN-CSP had ability to inhibit the cytopathic effect caused by VSV on WISH cells (1.2 × 10^8^ U/mg), and had similar anti-HBV activity compared with native IFN *α*2b (Figure 6). Meanwhile, the histology photomicrographs presented in Figure 7 confirmed the liver-targeting potentiality of IFN-CSP. These results indicated that fusing the *Plasmodium* region I-plus peptide with IFN *α*2b had almost no influence on structure of IFN *α*2b and *Plasmodium* region I-plus peptide.

In conclusion, a novel liver-targeting fusion interferon was successfully constructed by improved SOE-PCR strategy. The final yield of biologically active recombinant IFN-CSP was up to 270 mg/L culture in *E. coli* using pET-21b vector. The purified IFN-CSP exhibits anti-HBV activity and liver-targeting potentiality. IFN-CSP may be an excellent candidate as a liver-targeting anti-HBV agent.

## Figures and Tables

**Table 1:** Nucleotide sequences of oligonucleotides designed for assembly of IFN-CSP^a,b^.

Primers	Nucleotide sequences (from 5′ end to 3′ end)
IC-1	GGAATTC**CATATG** *TGTGATCTGCCTCAAA *
IC-2	*GTGCCAGGAGCATCAAGGT*CCTCCTGCTACCCAGGCTGT*GGGTTTGAGGCAGATCACA *
IC-3	*ACCTTGATGCTCCTGGCAC*AGATGAGGAGAATCTCTCTT*TTCTCCTGCTTGAAGGACA *
IC-4	*GGTTGCCAAACTCCTCCTG*GGGAAATCCAAAGTCATGTC*TGTCCTTCAAGCAGGAGAA *
IC-5	*CAGGAGGAGTTTGGCAACC*AGTTCCAAAAGGCTGAAACC*ATCCCTGTCCTCCATGAGA *
IC-6	*AGTCCTTTGTGCTGAAGAG*ATTGAAGATCTGCTGGATCA*TCTCATGGAGGACAGGGAT *
IC-7	*CTCTTCAGCACAAAGGACT*CATCTGCTGCTTGGGATGAG*ACCCTCCTAGACAAATTCT *
IC-8	*AGGCTTCCAGGTCATTCAG*CTGCTGGTAGAGTTCAGTGT*AGAATTTGTCTAGGAGGGT *
IC-9	*CTGAATGACCTGGAAGCCT*GTGTGATACAGGGGGTGGGG*GTGACAGAGACTCCCCTGA *
IC-10	*GGAAGTATTTCCTCACAGC*CAGAATGGAGTCCTCCTTCA*TCAGGGGAGTCTCTGTCAC *
IC-11	*GCTGTGAGGAAATACTTCC*AAAGAATCACTCTCTATCTG*AAAGAGAAGAAATACAGCC *
IC-12	*ATCTCATGATTTCTGCTCT*GACAACCTCCCAGGCACAAG*GGCTGTATTTCTTCTCTTT *
IC-13	*AGAGCAGAAATCATGAGAT*CTTTTTCTTTGTCAACAAAC*TTGCAAGAAAGTTTAAGAA *
IC-14	*CTTAATTTCTCGTTGTCTT*CCTTAC*TTCTTAAACTTTCTTGCAA *
IC-15	*AAGACAACGAGAAATTAAG*GAAACCAAAACATAAAAAA*TTAAAGCAACCAGCGGA *
IC-16	CCG**CTCGAG**ACCA*TCCGCTGGTTGCTTTAA *

^a^Restriction sites are bold.

^b^The italicized letters indicate the overlapped parts in IFN-CSP.
